# Advancing spinal research beyond COVID‐19

**DOI:** 10.1002/jsp2.1255

**Published:** 2023-03-27

**Authors:** Daisuke Sakai, Robert Leon, Mauro Alini

**Affiliations:** ^1^ Tokai University School of Medicine Graduate School of Medicine Orthopaedic Surgery 143 Shimokasuya Isehara Japan; ^2^ University of Pennsylvania McKay Orthopaedic Research Laboratory 114A Stemmler Hall, 36th Street and Hamilton Walk Philadelphia Pennsylvania US; ^3^ AO Foundation AO Research Institute Clavadelerstrasse 8 Davos Platz, Graubünden Switzerland

It is with deep emotion that finally, complete in‐person meetings have resumed beyond COVID‐19 in the field of spine research. The initial kick‐off started in November 6–10, 2022 with the success of ORS PSRS 6th International Spine Research Symposium held at Skytop Lodge located in picturesque Pocono Mountains, Pennsylvania. We appreciate that the ORS PSRS have agreed to put together a JOR Spine‐PSRS special issue, as has been done in pre‐pandemic times. We look forward to publishing cutting‐edge research articles from that event sometime later this year.

Then, finally came the ORS 2023 Annual Meeting in Dallas taking place from February 10–14, 2023. It was indeed a success, bringing over 2400 attendees world‐wide. It really touched everybody's hearts to be able to discuss in “Real” on topics people have warmed in their hands during days in the house. We were also pleased to be able to award the 2023 JOR Spine Early Career Award to Dr. Rebecca Wachs and Dr. Benjamin (Ben) Walter during the awards ceremony.

While we may not be entirely clear about the potential impact of future pandemic or disaster events, we can strongly agree with the fact that research activities do not strengthen or evolve to their maximum in an on‐line format. We all hope that this momentum and happiness of being together will bring peace and advancement of science in the field of spine research.

As for the journal metrics, it looks great and we forecast another milestone ahead, so we strongly encourage you to submit your best research to us!

Looking forward to working with you all (in person) again,

Dai, Rob and Mauro.

